# Comparative efficacy of gibberellic acid and melatonin seed priming on germination and biochemical responses of maize under drought stress

**DOI:** 10.3389/fpls.2026.1753761

**Published:** 2026-04-01

**Authors:** Km Babali Kumari, Apurba Pal, Prabhat Kumar, Shreya Sen, Rakesh Deo Ranjan, Kousik Atta, Ashutosh Kumar Srivastava

**Affiliations:** 1 Bihar Agricultural University, Sabour, India; 2 GLA University, Mathura, India; 3 Rani Lakshmi Bai Central Agricultural University, Jhansi, India

**Keywords:** drought stress, gibberellic acid, maize, melatonin, seed priming

## Abstract

Drought stress severely limits maize germination and early seedling growth by reducing germination percentage, seedling vigor, water uptake, and biomass, while prolonging mean germination time. This study evaluated the ameliorative effects of seed priming with gibberellic acid (GA_3_) and melatonin on 10 maize genotypes (CLO 2450, DHM 117, SML 1, VQL 1, SKM 1, SML 2, LM 13, SML 163, SML 165, and SML 28) under 10% PEG-induced osmotic stress. Germination traits, water relations, biomass accumulation, and biochemical parameters, including α-amylase activity, total soluble sugar, proline, total phenolic content, soluble protein, lipid peroxidation, and electrolyte leakage, were assessed at 1, 7, and 14 days. Drought stress markedly suppressed enzymatic activity, carbohydrate mobilization, osmolyte accumulation, and protein content, while increasing oxidative damage and membrane leakage. GA_3_ priming (100 ppm) partially restored germination and metabolic traits, whereas higher concentrations showed diminished recovery. Melatonin priming (200 µM) consistently improved germination (>92%), seedling length (~19–20 cm), water uptake (~88%–90%), biomass, and antioxidant defenses, while limiting lipid peroxidation and electrolyte leakage. Violin plot and heatmap analyses confirmed that melatonin treatments closely resembled control performance, whereas GA_3_ provided intermediate recovery.

## 1 Introduction

Maize (*Zea mays* L.) is one of the world’s most important cereal crops, leading global cereals in production volume and ranking third after rice and wheat in terms of cultivated area. It occupies approximately 197–201 million hectares worldwide and produces approximately 1.14–1.16 billion tonnes annually, reflecting its exceptional productivity and adaptability across diverse agro-ecological regions, including the United States, China, Brazil, sub-Saharan Africa, Latin America, and Asia (Gong et al., 2015; Erenstein et al., 2022; Food and Agriculture Organization of the United Nations, 2023; Wahengbam et al., 2024). Owing to its C_4_ photosynthetic efficiency, wide ecological plasticity, and versatile end uses, maize is a major contributor to global food security, livestock feed, biofuel production, and industrial raw materials, with grains rich in starch (~70%), protein (~9.9%), and oil (~4%) (Ranum et al., 2014). In India, maize production has reached 33.73 million tonnes from 9.95 million hectares, emphasizing the crop’s growing importance in meeting national food and feed demands (Department of Agriculture & Farmers Welfare, Ministry of Agriculture & Farmers Welfare, Government of India, 2023). However, maize productivity is increasingly challenged by abiotic stresses, particularly drought, which severely affects early growth stages such as seed germination and seedling establishment. Moisture stress disrupts water uptake, metabolic activity, and reserve mobilization, leading to poor vigor and yield penalties (Rafique, 2021; Gnawali and Subedi, 2021; Chen et al., 2025). Experimentally, drought stress is commonly simulated using polyethylene glycol (PEG-6000), which lowers cellular water potential and inhibits key enzymatic processes, including α-amylase-mediated starch hydrolysis essential for energy supply during germination (Aslam et al., 2015; Weisany et al., 2023; Luo et al., 2024). The above situation triggers the production of reactive oxygen species (ROS), which are accumulated, causing the peroxidation of lipids, damage to the membranes, and cellular oxidative stress, which in turn leads to the reduction in the normal seed germination process (down to <50% in extreme conditions), radicle growth, and small plant (seedling) biomass (Farooq et al., 2011; Yang et al., 2023; Pongtip et al., 2024; Velicevici et al., 2025). Climate change exacerbates these events, with projections indicating a 20%–30% rise in drought frequency by 2050; thus, it is necessary to adopt the strategies that can assure the maize establishment (Tayyab et al., 2020; Shemi et al., 2021; Li et al., 2023; Wang M. et al., 2025).

Priming of seeds, which is the pre-sowing hydration or chemical treatment that activates metabolism prior to germination without radicle protrusion, is a cheaper solution to bring about the uniformity of germination, speed of emergence, and stress tolerance in maize (Paparella et al., 2015). Different priming methods, including hydro-, osmo-, halo-, nutri-, chemical, bio-, nano-, and magneto-priming, have been shown to be effective, but the methods based on phytohormones like gibberellic acid (GA_3_) and melatonin are superior due to their specific interference with the growth and defense pathways (Nada and Hamza, 2018; Gnawali and Subedi, 2021; Rajora et al., 2022; Luo et al., 2024). GA_3_ priming speeds up the germination of maize during drought by activating the α-amylase and dehydrogenase for starch breakdown and energy supply; thus, cell elongation and radicle emergence are brought about (Gnawali and Subedi, 2021; Luo et al., 2024; Chen et al., 2025). However, melatonin priming is unbeatable in antioxidative defense; it not only scavenges ROS but also ensures the stability of membranes, modulates the hormones, and thus lessens the oxidative damage in maize seedlings under stress (Zhang et al., 2013; Lv et al., 2021). Melatonin application in drought-stressed maize is a way to relieve stress from ROS bursts, increase aquaporin and GA synthesis, and decrease ABA, leading to improved osmotic adjustment and higher root hydraulic conductivity under PEG (Wang et al., 2024). The two, however, support maize’s resilience differently: GA_3_ by stimulating a metabolic pathway and melatonin by maintaining a healthy redox balance. They, however, cause different things to happen: GA_3_ speeds up growth and, thereby, the risk of ROS accumulation due to high metabolism, while melatonin brings together the ROS signaling, hormonal rebalancing (for example, the GA/ABA ratio), and wide-ranging protection against drought, salinity, heat, and even floods (Kakar et al., 2023; Gelaw and Sanan-Mishra, 2024). The effectiveness of GA_3_ in maize under drought has been well established (Khan et al., 2020; Muhammad et al., 2023), but no assessments with melatonin in direct comparison are available, especially concerning genotype-specific responses and osmotic stress during germination. There are reports of genotypic variability in GA_3_ priming results in different maize lines (Kakar et al., 2023; Badr et al., 2020), but the same has not yet been studied for melatonin. This research fills these voids through the direct comparison of GA_3_ and melatonin priming with selected maize genotypes under PEG-induced drought stress.

## 2 Materials and methods

### 2.1 Seed material and experimental setup

A laboratory experiment was carried out at the Seed Physiology Lab, Department of Plant Physiology and Biochemistry, BAU, Sabour. Standardization of the concentrations of GA_3_ and melatonin effective for pre-sowing seed priming ameliorated moisture stress. For that, four genetically diverse maize genotypes (CLO 2450, DHM 117, SML 1, and VQL 1) were initially selected for standardizing priming agent concentrations under drought stress conditions. Seeds were obtained from the All India Coordinated Research Project (AICRP)-Maize, BAU, Sabour, ensuring uniformity in size and viability (>95% germination under control conditions). Standardization experiments were conducted in a controlled laboratory environment at 25 °C ± 2 °C with a 16/8-hour light/dark photoperiod (300 µmol m^−2^ s^−1^ light intensity). A completely randomized design (CRD) was used with three replicates per treatment, each replicate consisting of 50 seeds per genotype. Seeds were surface-sterilized with 1% sodium hypochlorite for 5 min, rinsed thoroughly with distilled water, and air-dried to their original moisture content.

### 2.2 Seed priming and drought stress simulation

Priming solutions were prepared using GA_3_ at concentrations of 50, 100, and 200 ppm (designated DSGA50, DSGA100, and DSGA200, respectively) and melatonin at 50, 100, and 200 µM (DSMEL50, DSMEL100, and DSMEL200, respectively). Priming was performed by soaking seeds in the respective solutions for 12 hours at 25 °C in the dark, followed by air-drying to restore the original moisture content. Drought stress was simulated using 10% polyethylene glycol (PEG-6000, w/v) in the germination medium, corresponding to an osmotic potential of approximately −0.5 MPa (Khan et al., 2019, 2020; Badr et al., 2020; Bukhari et al., 2021; Mustamu et al., 2023; Muhammad et al., 2023; Zhang et al., 2024). Non-stressed controls (C) were germinated in distilled water. Seeds were placed on two layers of Whatman No. 1 filter paper in 15-cm Petri dishes and moistened with 10 mL of either distilled water (control) or 10% PEG-6000 solution [drought stress (DS)]. Petri dishes were sealed and incubated under the described conditions. Germination was recorded daily for 7 days, with a seed considered germinated when the radicle emerged ≥2 mm.

### 2.3 Germination parameters

The following germination parameters were calculated:

- Germination percentage (GP): GP = (Number of germinated seeds/Total seeds) × 100 (International Seed Testing Association, 2019).
- Germination speed (GS): GS = Σ(Ni/Ti), where Ni is the number of seeds germinated on day Ti (Maguire, 1962).
- Mean germination time (MGT): MGT = Σ (Ti × Ni)/ΣNi, where Ti is the day of germination, and Ni is the number of seeds germinated on that day (Ellis & Roberts, 1981).
- Mean daily germination (MDG): MDG = GP/Number of days to final count (Czabator, 1962).
- Peak value (PV): PV = Maximum number of seeds germinated on any single day/Day of peak germination (Czabator, 1962).
- Germination value (GV): GV = PV × MDG (Czabator, 1962).

After 7 days, 10 seedlings per replicate were randomly selected to measure seedling length (radicle + plumule; cm), fresh weight (g), and dry weight (g) after oven-drying at 70 °C for 48 hours. Water uptake (%) was calculated as [(Fresh weight − Dry weight)/Dry weight] × 100 (International Seed Testing Association, 2019).

### 2.4 Expanded genotype evaluation

Following standardization, 10 maize genotypes, viz. CLO 2450, DHM 117, SML 1, VQL 1, SKM 1, SML 2, LM 13, SML 163, SML 165, and SML 28, were selected from the AICRP-Maize, BAU, Sabour. Standardized concentrations of GA_3_ and melatonin were applied as priming agents. Primed and non-primed seeds were subjected to osmotic stress using 10% PEG-6000. Fifty seeds per replicate were placed on Whatman No. 1 filter paper in 90-mm Petri dishes and moistened with 5 mL of 10% PEG-6000 solution for drought stress or 5 mL of distilled water for controls. Petri dishes were sealed and incubated as described. Germination was monitored daily for 14 days, with radicle emergence (≥2 mm) as the germination criterion. Germinating seeds were sampled at 1, 7, and 14 days post-stress exposure for biochemical analyses. Embryo axes (radicle and plumule) were excised, snap-frozen in liquid nitrogen, and stored at −80 °C until analysis. Fresh and dry weights of the embryo axes were recorded before and after oven-drying at 60 °C for 48 hours to assess water content and biomass accumulation.

### 2.5 Biochemical analysis

#### 2.5.1 Amylase activity

Amylase activity was assayed by the 3,5-Dinitrosalicylic acid (DNS) method (Bernfeld, 1955). Frozen embryo axes (0.1 g) were homogenized in 1 mL of 50 mM sodium phosphate buffer (pH 7.0) containing 1% Polyvinylpyrrolidone (PVP), centrifuged (12,000 × *g*, 15 min, 4 °C), and the supernatant was used as the enzyme extract. The assay mixture (0.5 mL extract + 0.5 mL 1% soluble starch in buffer) was incubated at 37 °C for 30 min. Reactions were terminated with 1 mL DNS reagent and boiled for 5 min, and absorbance was measured at 540 nm (UV-1800, Shimadzu Corporation, Kyoto, Japan). Activity was expressed as µg maltose min^−1^ g^−1^ FW.

#### 2.5.2 Total soluble sugars

Total soluble sugar (TSS) was estimated using the anthrone method (Yemm and Willis, 1954). Embryo axes (0.1 g) were extracted with 5 mL of 80% ethanol (80 °C, 30 min) and centrifuged (10,000 × *g*, 10 min). The supernatant was reacted with 4 mL of 0.2% anthrone in 95% H_2_SO_4_, boiled for 10 min, and cooled, and the absorbance was read at 620 nm. Values were expressed as mg g^−1^ FW (glucose equivalents).

#### 2.5.3 Proline content

Proline was determined following Bates et al. (1973). Samples (0.1 g) were homogenized in 5 mL of 3% sulfosalicylic acid and centrifuged (10,000 × *g*, 10 min), and 2 mL of supernatant was mixed with equal volumes of acid ninhydrin and glacial acetic acid. After incubation (100 °C, 1 hour) and cooling, 4 mL toluene was added; absorbance of the toluene phase was read at 520 nm. Results were expressed as µM g^−1^ FW.

#### 2.5.4 Total soluble protein

Proteins were quantified using the Bradford method (Bradford, 1976). Embryo axes (0.1 g) were homogenized in 1 mL of 50 mM Tris-HCl (pH 7.5) with 1 mM EDTA and 1% PVP and centrifuged (12,000 × *g*, 15 min, 4 °C), and 0.1 mL supernatant was mixed with 5 mL Bradford reagent. Absorbance was recorded at 595 nm, and protein content was expressed as mg g^−1^ FW (Bovine Serum Albumin (BSA) equivalents).

#### 2.5.5 Total phenolic content

Total phenolic content (TPC) was determined using the Folin–Ciocalteu reagent (Singleton and Rossi, 1965). Homogenates of embryo axes (0.1 g in 5 mL 80% methanol) were centrifuged (10,000 × *g*, 10 min). Supernatant (0.5 mL) was reacted with 2.5 mL of 10% Folin–Ciocalteu and 2 mL of 7.5% sodium carbonate and incubated 30 min at 40 °C, and absorbance was read at 765 nm. Results were expressed as mM GAE g^−1^ FW.

#### 2.5.6 Lipid peroxidation (TBARS)

Lipid peroxidation was estimated as Thiobarbituric Acid Reactive Substances (TBARS) (Heath and Packer, 1968). Embryo axes (0.1 g) were homogenized in 5 mL of 0.1% Trichloroacetic Acid (TCA) and centrifuged (10,000 × *g*, 10 min); 1 mL supernatant was mixed with 4 mL of 0.5% Thiobarbituric Acid (TBA) in 20% TCA, heated (95 °C, 30 min), cooled, and centrifuged. Absorbance was measured at 532 nm (corrected at 600 nm). Values were expressed as µM g^−1^ FW.

#### 2.5.7 Electrolyte leakage

Membrane damage was assessed using electrolyte leakage (Lutts et al., 1996). Embryo axes (0.1 g) were incubated in 10 mL deionized water at 25 °C for 24 hours, and the conductivity (EC1) was recorded. Samples were then boiled (20 min) and cooled, and conductivity was measured again (EC2). Leakage was expressed as (EC1/EC2) × 100 (%).

### 2.6 Statistical analysis

All experiments followed a CRD with three biological replicates per treatment. Data were analyzed using one-way ANOVA to evaluate the effects of drought stress and seed priming (GA_3_ and melatonin) on germination, seedling growth, and biochemical traits. Multiple comparisons were performed using Tukey’s honestly significant difference (HSD) test (p < 0.05). Results are expressed as mean ± SE, with different letters indicating significant differences. Data visualization included violin plots (trait distribution and variability), heatmaps (treatment clustering and priming effects), and interaction plots (treatment × time responses of key biochemical variables). Statistical analyses and graphics were carried out in the R software (v.4.2.2) using suitable packages for ANOVA, *post hoc* testing, and visualization.

## 3 Result

### 3.1 Standardization of concentration of GA3 effective for pre-sowing seed priming to ameliorate drought stress

Data in **Table 1** represent the optimized concentrations of GA_3_ and melatonin used for seed priming prior to sowing, aimed at enhancing germination responses in maize genotypes under osmotic stress conditions. Germination percentage exhibited significant variation among genotypes and treatments. Under control conditions, germination remained consistently high (>96%) across all genotypes, with DHM 117 (97.33%) and VQL 1 (97.20%) showing slightly better performance than CLO 2450 (97.18%) and SML 1 (96.93%). Imposition of 10% PEG drought stress drastically reduced germination, with values declining to nearly 68%–69% across genotypes, reflecting a ~28%–30% reduction compared to their respective controls. Exogenous GA_3_ and melatonin efficiently alleviated this inhibition. At 100 ppm GA_3_, germination was restored to as high as 89.18% in DHM 117 and 88.92% in CLO 2450, with marginally lower values in SML 1 (86.92%) and VQL 1 (88.57%). However, at 200 ppm, germination declined slightly, suggesting possible supra-optimal inhibition of hormonal balance. In contrast, melatonin was more effective, producing a steady, concentration-dependent restoration. At GA 200 µM, germination was restored close to control values, reaching 93.27% in DHM 117 and 92.84% in CLO 2450. The mean germination percentages confirmed DHM 117 as the most responsive genotype (90.48%), followed by CLO 2450 (89.95%), VQL 1 (89.70%), and SML 1 (88.36%). Statistical analysis indicated significant differences, with critical differences at the 0.05 level ranging from 2.81 to 4.29, suggesting true genotypic × treatment effects. Germination speed followed similar trends. Control seeds showed the highest rates (16.2–16.4), while drought stress reduced speed by more than 50% (as low as 6.18 in DHM 117 and 6.04 in SML 1). Exogenous treatments enhanced germination velocity, with maximum recovery achieved under melatonin priming at 200 µM (12.65 in VQL 1; 12.64 in CLO 2450), whereas DHM 117 also improved markedly (12.28). GA_3_ exhibited a moderate recovery effect, with 100 ppm supporting significantly higher rates (11.82–12.67). Stress significantly prolonged mean germination time, rising from 3.7–3.8 days (control) to >5.0 days under PEG stress. Both GA_3_ and melatonin markedly shortened MGT, with the lowest times recorded at 100 µM melatonin (3.80–4.10 days) and 100 ppm GA_3_ (4.25–4.45 days). Peak values, reflecting germination efficiency, declined drastically under stress (4.8–5.2) compared to controls (8.0–8.3). Both GA_3_ and melatonin increased peak values considerably, with maximum peaks observed at 100 µM melatonin (MEL) (7.9–9.1) and 100 ppm GA_3_ (7.1–7.5), again highlighting the superiority of melatonin at enhancing uniform germination under osmotic stress. A similar pattern was observed in MDG. Control values were the highest (4.50–4.63) but were halved under drought (2.17–2.29). Priming agents improved MDG to 3.7–4.2 across treatments, with melatonin at 200 µM supporting the highest recovery (4.2 in VQL 1 and SML 1). PEG stress reduced water uptake considerably (69%–74%) compared to controls (90%–92%). GA_3_ and melatonin restored water absorption in seeds, with maximum uptakes achieved at melatonin 200 µM (88%–90%). DHM 117 (90.12%), VQL 1 (89.90%), and CLO 2450 (89.63%) exhibited higher water flux into seed tissues under melatonin treatment, indicating improved osmotic adjustment. Seedling length was also markedly reduced under drought, decreasing from 20.25 cm in the control to 13.15 cm. GA_3_ priming at 100 ppm (T4G2) increased length to 17.79 cm (35.4% improvement), with 50- and 200-ppm treatments achieving 15.66 and 16.75 cm, respectively. Melatonin priming further enhanced elongation, with 200 µM (T8M3) achieving 18.96 cm (44.2% over stress), and 100 and 50 µM improving length to 17.78 and 16.92 cm, respectively. Fresh weight of seedlings declined from 2.678 g in the control to 1.933 g under drought stress, reflecting reduced water content, nutrient mobilization, and metabolic activity. GA_3_ priming at 100 ppm (T4G2) partially alleviated this effect, increasing fresh weight to 2.462 g (18% over stress), while 50 ppm (T3G1) and 200 ppm (T5G3) treatments reached 2.282 and 2.336 g, respectively. Melatonin priming was more effective, with 200 µM (T8M3) restoring fresh weight to 2.540 g (23.9% over stress), and 100 µM (T7M2) and 50 µM (T6M1) increasing it to 2.468 and 2.376 g, respectively. Dry weight of seedlings decreased from 0.139 g in the control to 0.105 g under drought stress. GA_3_ at 100 ppm (T4G2) elevated dry weight to 0.137 g (24.2% over stress), with 50 and 200 ppm increasing it to 0.122 and 0.126 g, respectively. Melatonin at 200 µM (T8M3) restored dry weight to 0.137 g (30% increase), while 100 and 50 µM improved it to 0.132 and 0.128 g, respectively.

**Table 1:** Effect of 10% PEG-6000-induced drought stress on germination and seedling traits in four maize genotypes.

Traits	Genotype	C	DS	DSGA50	DSGA100	DSGA200	DSMEL50	DSMEL100	DSMEL200	MEAN	C.D. (0.05)	MSE	SED	C.V.
Germination percentage (%)	CLO 2450	97.180a	69.910f	80.740e	88.920bcd	84.930de	87.180cd	89.630bc	92.840ab	89.950	2.8126	2.6404	1.3268	1.88
	DHM 117	97.330a	68.750e	81.620d	89.180bc	85.910cd	87.540bcd	90.630abc	93.270ab	90.476	4.2347	5.9855	1.9976	2.82
	SML 1	96.930a	68.640f	79.680e	86.920bcd	82.810de	84.470cd	88.320bc	89.820b	88.360	2.8495	2.7101	1.3441	1.94
	VQL 1	97.200a	69.170e	80.340d	88.570abc	84.660 cd	86.820bcd	89.420abc	92.380ab	89.699	4.2885	6.1387	2.023	2.89
Germination speed	CLO 2450	16.320a	6.940f	11.260e	12.670c	11.820de	11.810de	12.280cd	13.650b	13.391	0.4562	0.2152	0.4562	2.17
	DHM 117	16.210a	6.180f	9.310e	10.800cd	10.040de	10.100cde	11.000c	12.600b	12.159	0.5673	0.1074	0.2676	3.03
	SML 1	16.000a	6.400f	10.500e	12.800bc	11.600d	12.100cd	12.900bc	13.500b	13.300	0.5191	0.0899	0.5191	2.50
	VQL 1	16.400a	6.800e	11.000d	12.400c	11.700cd	11.600cd	12.100c	13.500b	13.263	0.6578	0.1444	0.3103	3.19
Mean germination time	CLO 2450	3.750cd	5.150a	4.650ab	4.300bcd	4.450bc	4.550bc	4.100bcd	3.850d	4.300	0.3473	0.0403	0.1638	4.57
	DHM 117	3.650cd	5.280a	4.620b	4.180bcd	4.380bc	4.400bc	4.050bcd	3.710d	4.205	0.3901	0.0508	0.184	5.21
	SML 1	3.800c	5.300a	4.800ab	4.100c	4.400bc	4.300bc	4.200bc	4.000c	4.300	0.3844	0.0493	0.1813	5.05
	VQL 1	3.780bc	5.180a	4.680ab	4.250bc	4.420bc	4.480bc	4.100bc	3.880c	4.296	0.4143	0.0573	0.1954	5.47
Peak value	CLO 2450	8.200a	5.100e	6.300d	7.100bc	6.700cd	6.900bc	7.400b	8.100a	7.488	0.3613	0.1704	0.1707	2.98
	DHM 117	8.330a	4.830e	5.830d	7.100c	6.500cd	6.700c	6.830c	7.900b	7.315	0.4381	0.0641	0.2067	3.73
	SML 1	8.000a	5.000e	6.300d	7.200bc	6.600cd	6.900bcd	7.000bc	7.500b	7.313	0.4158	0.0577	0.1961	3.51
	VQL 1	8.100a	5.200e	6.200d	7.200c	6.800cd	6.900cd	7.300bc	8.000ab	7.450	0.4891	0.0798	0.2307	4.05
Mean daily germination	CLO 2450	4.533a	2.233d	3.233c	3.667bc	3.433c	3.533c	3.733bc	4.200b	3.983	0.3358	0.0376	0.1584	5.37
	DHM 117	4.637a	2.293d	2.970c	3.330c	3.197c	3.247c	3.507bc	3.967b	3.811	0.3591	0.043	0.1694	6.04
	SML 1	4.500a	2.167d	3.167c	3.667bc	3.467bc	3.433bc	3.567bc	3.800b	3.888	0.3681	0.0452	0.1736	6.07
	VQL 1	4.533a	2.233d	3.200c	3.700bc	3.433c	3.500c	3.700bc	4.200ab	3.975	0.3885	0.0504	0.1833	6.25
Water uptake (%)	CLO 2450	91.300a	70.800e	79.100d	85.800bc	83.200cd	83.600c	85.400bc	89.600ab	86.288	2.6645	2.3696	1.2569	1.84
	DHM 117	92.780a	74.200d	80.870cd	86.990abc	84.000bc	84.600bc	86.430abc	90.120ab	87.446	4.1297	5.6925	1.9481	2.81
	SML 1	90.900a	69.800d	78.300c	82.900b	80.400bc	81.000bc	82.500bc	84.300b	84.025	2.7514	2.5268	1.2979	1.96
	VQL 1	91.500a	70.300d	78.700c	85.300abc	82.600bc	83.000abc	85.100ab	89.300abc	86.000	4.1713	5.8078	1.9677	2.90
Total seedling length (cm)	CLO 2450	20.200a	13.000f	15.800e	17.900bc	16.700de	17.000cd	18.000b	19.700a	18.313	0.5809	0.1126	0.2740	1.94
	DHM 117	20.880a	13.610d	15.550c	18.260b	17.010b	17.100b	18.120b	19.760a	18.570	0.8669	0.2509	0.409	2.85
	SML 1	19.800a	12.800e	15.700d	17.200bc	16.500cd	16.800bc	17.100bc	17.800b	17.713	0.6512	0.1415	0.3072	2.25
	VQL 1	20.100a	13.200e	15.600d	17.800bc	16.800cd	16.800cd	17.900bc	18.600ab	18.088	0.908	0.2752	0.4283	3.07
Total seedling fresh weight (mg)	CLO 2450	2.615 ab	1.985 d	2.404 c	2.568abc	2.446bc	2.584 ab	2.664 a	2.668 a	484.500	0.1092	0.004	0.0515	2.53
	DHM 117	2.730a	2.033d	2.303c	2.520b	2.411bc	2.288c	2.453b	2.480b	495.125	0.0874	0.0026	0.0412	2.1
	SML 1	2.685a	1.838d	2.177bc	2.261bc	2.135c	2.229bc	2.207bc	2.291b	473.125	0.0787	0.0021	0.0371	2.04
	VQL 1	2.668ab	1.876e	2.245d	2.497abcd	2.353cd	2.404a	2.550abc	2.720bcd	480.375	0.1784	0.0106	0.0842	4.27
Total seedling dry weight (mg)	CLO 2450	0.138ab	0.104d	0.127c	0.136bc	0.131bc	0.133bc	0.139a	0.146ab	136.138	0.0058	0.000	0.0027	2.53
	DHM 117	0.142a	0.112e	0.121d	0.132bc	0.126cd	0.127cd	0.128cd	0.138ab	132.200	0.0047	0.000	0.0022	2.10
	SML 1	0.137a	0.102e	0.114d	0.120bcd	0.117cd	0.118bcd	0.121bc	0.124b	123.625	0.0042	0.000	0.002	2.04
	VQL 1	0.137a	0.103b	0.125a	0.135a	0.130a	0.132a	0.138a	0.139a	134.250	0.0096	0.000	0.0045	4.26

Values are means of three replicates. Different letters within a column indicate significant differences at p < 0.05 according to Tukey’s honestly significant difference (HSD) test.

Leveled as follows: C, control; DS, drought stress (10% PEG-6000); DSGA50, GA_3_ 50 ppm + DS; DSGA100, GA_3_ 100 ppm + DS; DSGA200, GA_3_ 200 ppm + DS; DSMEL50, melatonin 50 µmol + DS; DSMEL10, melatonin 100 µM + DS; DSMEL200, melatonin 200 µmol + DS.

C.D. (0.05), critical difference at 5% level of significance; MSE, mean square error; SED, standard error of difference; C.V., coefficient of variation.

#### 3.1.1 Integrative violin plot and heatmap approach to determine effective melatonin and GA_3_ priming for drought tolerance in maize

**Figure 1:**
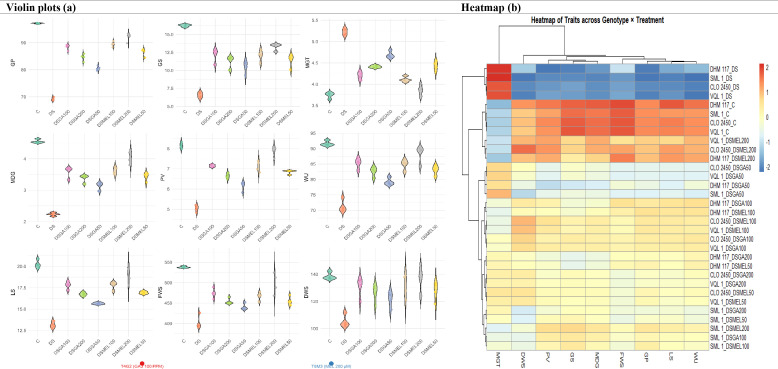
Violin plots **(a)** and heatmap **(b)** representing germination and seedling traits of maize genotypes under control **(c)**, drought stress (DS; 10% PEG-6000), and priming with gibberellic acid (GA_3_) (DSGA50, 50 ppm; DSGA100, 100 ppm; DSGA200, 200 ppm) or melatonin (DSMEL50, 50 µM; DSMEL100, 100 µM; DSMEL200, 200 µmol). Traits assessed include germination percentage (GP), germination speed (GS), mean germination time (MGT), mean daily germination (MDG), peak value (PV), water uptake (WU), seedling length (LS), seedling fresh weight (FWS), and seedling dry weight (DWS). In violin plots, horizontal width reflects data density, highlighting treatment effects on trait distributions. Drought stress markedly reduced germination efficiency, water uptake, and seedling vigor, while ligand priming alleviated these effects: GA_3_ showed maximum recovery at 100 ppm, and melatonin, particularly at 200 µM, was most effective, restoring values close to control levels. The heatmap **(b)** illustrates comparative performance across traits, with a red-to-blue scale indicating high to low values and cyan highlights marking the best-performing treatments.

The violin plots (**Figure 1a**) clearly illustrate treatment-wise variations in germination and seedling traits. Under control conditions (C), all traits—GP, GS, MGT, PV, MDG, water uptake (WU), total seedling length (LS), total seedling fresh weight (FWS), and total seedling dry weight (DWS)—were at their highest levels with rapid germination and vigorous seedlings, whereas DS caused sharp declines in GP, GS, WU, and biomass, while prolonging MGT, thereby confirming severe inhibition. GA_3_ priming improved performance in a concentration-dependent manner, with 100 ppm (T4G2) achieving the best recovery across germination traits; however, further increase to 200 ppm (T5G3) showed a slight decline. Melatonin exhibited a stronger and more consistent protective effect, with progressive improvements from T6M1 (50 µM) to T8M3 (200 µM), where most parameters, including GP (>92%), GS, water uptake (~89%–90%), seedling length (~19–20 cm), and dry weight, were restored close to control levels. Distributions were narrower in melatonin treatments, indicating uniform recovery compared to DS. Overall, the plots highlight melatonin (200 µM) as the most effective, followed by GA_3_ at 100 ppm, in mitigating drought-induced germination and seedling inhibition. The heatmap (**Figure 1b**) of germination and seedling traits revealed clear clustering of treatments. Drought stress was grouped separately, showing the lowest values across all parameters. Control treatments clustered with melatonin 200 µM (T8M3), highlighting their similarity in maintaining high germination percentage, water uptake, seedling length, and biomass. GA_3_ treatments showed intermediate recovery, with 100 ppm (T4G2) and melatonin 100 µM (T7M2) grouping closer to high-performance clusters, while GA_3_ at 200 ppm (T5G3) and 50 ppm (T3G1) were less effective. Overall, melatonin, particularly at 200 µM, was consistently highlighted as the top-performing treatment across traits, followed by GA_3_ at 100 ppm, confirming melatonin’s superior role in drought stress mitigation.

### 3.2 Amelioration effect of seed priming on biochemical parameters in germinating seeds of 10 maize genotypes

**Figure 2:**
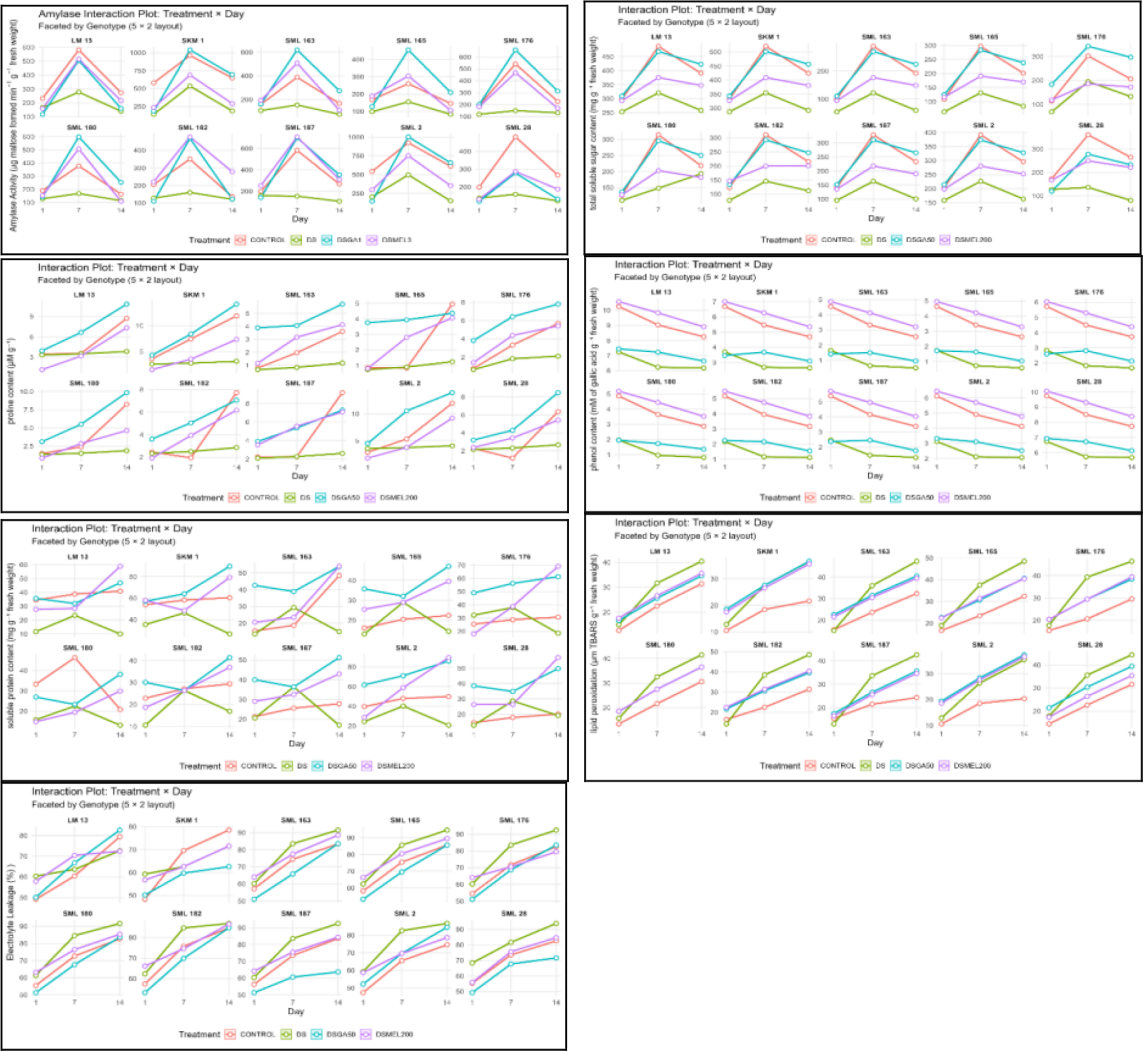
Interaction plots depicting the effects of seed priming on key biochemical parameters in 10 maize genotypes (SML 2, SML 13, SML 28, SML 163, SML 165, SML 176, SML 180, SML 182, and SML 187) across three time points (1, 7, and 14 days). Parameters include amylase activity (µg maltose formed min^−1^ g^−1^ FW), total soluble sugar (TSS; mg g^−1^ FW), proline content (µmol g^−1^), total phenolic content (mM gallic acid g^−1^ FW), soluble protein (mg g^−1^ FW), lipid peroxidation (µmol TBARS g^−1^ FW), and electrolyte leakage (%). The consistent separation of lines indicates significant treatment × time interactions (p < 0.05), highlighting the differential modulation of biochemical responses by seed priming over the germination period.

Following preliminary standardization, 10 maize genotypes, CLO 2450, DHM 117, SML 1, VQL 1, SKM 1, SML 2, LM 13, SML 163, SML 165, and SML 28, were selected from the AICRP-Maize, BAU, Sabour. Seed priming was performed using standardized concentrations of GA_3_ (100 µM) and melatonin (200 µM). Primed and non-primed seeds were exposed to osmotic stress simulated by 10% PEG-6000. Interaction plots (**Figure 2**) illustrate the effects of seed priming on key biochemical parameters in 10 maize genotypes assessed at three time points (1, 7, and 14 days).

Under drought stress (10% PEG-6000), α-amylase activity (µg maltose formed min^−1^ g^−1^ FW) in germinating seeds was significantly suppressed, declining from 545.07 µg at day 7 in controls to 241.17 µg under stress. GA_3_ priming substantially restored enzymatic activity, exceeding control levels in several genotypes. SKM 1 and SML 2 exhibited peak activities of 1,041.69 and 1,003.37 µg, respectively, at day 7, indicating enhanced starch hydrolysis and metabolic activation. Melatonin priming moderately increased amylase activity, particularly at day 14, suggesting sustained hydrolytic metabolism through antioxidant-mediated regulation. Statistical analyses revealed significant treatment effects (C.V. <3%), confirming reliable differentiation among treatments. TSS (mg g^−1^ FW) increased from day 1 to day 7 under control conditions, followed by slight stabilization or decline by day 14, reflecting normal carbohydrate mobilization. Drought stress severely reduced TSS across all genotypes, with pronounced declines by day 14. GA_3_ priming under stress restored and enhanced TSS, surpassing control values in top-performing genotypes (SKM 1, LM 13, and SML 2) by day 14, indicating efficient hydrolytic enzyme activation. Melatonin priming led to moderate but steady TSS elevation over 14 days, likely via the stabilization of carbohydrate metabolism through redox homeostasis. Significant treatment × genotype interactions and low variability metrics corroborate these trends, emphasizing GA_3_’s dominant role in sugar mobilization and melatonin’s contribution to sustained metabolic activity.

Proline content (µM g^−1^) under control conditions progressively increased over 14 days, peaking in SML 2 and SKM 1 (up to 12.00 µM g^−1^). Drought stress markedly reduced proline accumulation (mean 2.556 µM g^−1^ at day 14). Seed priming with GA_3_ significantly enhanced proline levels under stress, exceeding control values in SKM 1 (14.115 µM g^−1^) and SML 2 (13.985 µM g^−1^), suggesting stimulation of osmotic adjustment via hormonal and redox-mediated pathways. Melatonin priming moderately improved proline content, reaching 9.191 µM g^−1^ in SML 2 at day 14. Genotypic and treatment effects were significant (C.D. 0.179–0.266; S.E.d. 0.085–0.148; C.V. 2.08%–4.10%), demonstrating the efficacy of both priming agents in mitigating drought-induced proline depletion. TPC (mM gallic acid g^−1^ FW) decreased under drought stress across all genotypes, with the most sensitive genotypes (SML 165, SML 163) exhibiting the lowest levels by day 14 (1.86 mM). GA_3_ priming partially restored TPC, while melatonin priming fully recovered and exceeded control levels (up to 6.64 mM at day 1), indicating robust antioxidative protection. Genotypes SML 2 and LM 13 displayed the highest TPC under melatonin treatment, reflecting enhanced phenolic biosynthesis and oxidative stress mitigation. Statistical analyses confirmed the significance and precision of these observations. Soluble protein content (mg g^−1^ FW) increased under control conditions from 27.7 to 35.1 mg g^−1^ by day 14. Drought stress reduced protein levels (mean 33.3 mg g^−1^ at day 14). GA_3_ priming substantially enhanced protein accumulation, peaking at 56.5 mg g^−1^ in SKM 1, SML 2, and LM 13, whereas melatonin induced a moderate, sustained increase (mean 40.0 mg g^−1^), highlighting its role in protein stabilization. Treatment effects were significant with low variability (C.V. 1.6%–3.9%).

Lipid peroxidation (µm TBARS g^−1^ FW) increased under drought stress, with SML 165 and LM 13 showing peak levels of 38.241 and 34.126 µm, respectively, at day 14. GA_3_ priming further elevated TBARS (mean 37.939 µm). Conversely, melatonin priming limited lipid peroxidation (mean 37.421 µm), particularly in SKM 1 and SML 176, consistent with its ROS-scavenging and antioxidative enzyme-inducing functions. Statistical parameters confirmed significant treatment and genotype effects. Electrolyte leakage (%) increased over time under all treatments. Drought stress elevated leakage to 88.41% by day 14, particularly in SML 165 and SML 182. GA_3_ priming moderately reduced leakage at day 7 but remained elevated by day 14 (mean 82.17%). Melatonin effectively reduced membrane damage (mean 79.47%), improving stability in SKM 1 (71.56%) and LM 13 (77.83%). Statistical parameters [CD 2.44; SE(m) 0.85; C.V. 1.27%–3.37%] indicated significant and consistent treatment effects. Melatonin exhibited superior protection against drought-induced membrane damage compared to GA_3_.

#### 3.2.1 Comparative efficacy of gibberellic acid and melatonin seed priming on biochemical responses of maize under drought stress

**Figure 3:**
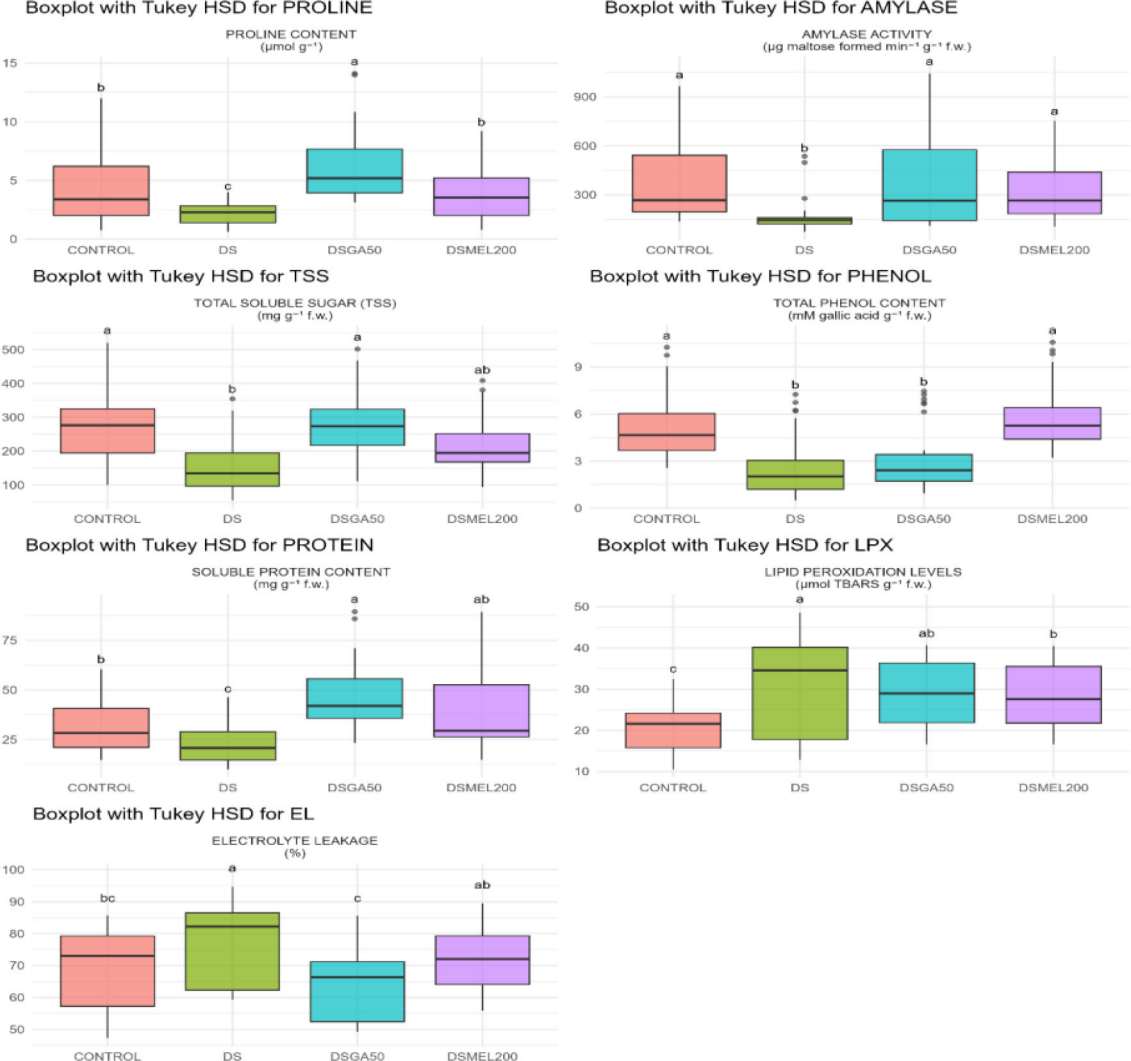
Box plots showing the effects of drought stress (DS) and melatonin (DSMEL200) or gibberellic acid (GA_3_) (DSGA50) priming on biochemical parameters in maize during early seedling stage. Values represent means ± SE; different letters indicate significant differences (p < 0.05).

The box plot (**Figure 3**) illustrates the comparative effects of GA_3_ (50 ppm) and MEL (200 µM) on key biochemical parameters in DS maize relative to the control and DS-alone groups. Drought stress markedly reduced proline (~6 to ~2 μmol g^−1^), amylase activity (~450 to ~20 μg maltose min^−1^ g^−1^ FW), total soluble sugars (~320 to ~140 mg g^−1^ FW), total phenols (~4.5 to ~1.8 mM gallic acid g^−1^ FW), and soluble protein (~38 to ~18 mg g^−1^ FW), while lipid peroxidation (~12 to ~42 μmol g^−1^ FW) and electrolyte leakage (~72% to ~92%) increased, indicating severe oxidative stress and membrane damage. GA_3_ priming (DSGA50) restored or exceeded control levels for proline (~10 μmol g^−1^), amylase (~500 μg min^−1^ g^−1^ FW), TSS (~320 mg g^−1^ FW), and protein (~48 mg g^−1^ FW); moderately increased phenols (~2.8 mM g^−1^ FW); and reduced lipid peroxidation (~35 μmol g^−1^ FW) and electrolyte leakage (~62%). Melatonin (DSMEL200) fully recovered phenol content (~4.5 mM g^−1^ FW); substantially improved protein (~43 mg g^−1^ FW), amylase (~400 μg min^−1^ g^−1^ FW), and TSS (~240 mg g^−1^ FW); modestly increased proline (~5 μmol g^−1^); and more effectively suppressed lipid peroxidation (~30 μmol g^−1^ FW), although less so for electrolyte leakage (~75%). GA_3_ primarily enhanced osmolyte accumulation and enzymatic activity for osmotic adjustment, while melatonin strengthened antioxidant defenses, highlighting their complementary mechanisms in mitigating drought stress.

## 4 Discussion

### 4.1 Standardization of gibberellic acid and melatonin seed priming in mitigating drought-induced inhibition of maize germination and early seedling growth

Drought stress is well established as one of the most critical abiotic constraints limiting maize seed germination and seedling establishment, primarily through osmotic inhibition, impaired enzyme activity, reduced mobilization of reserves, and oxidative damage (Farooq et al., 2009; Sheoran et al., 2022; Singh et al., 2023a). The present study demonstrated that drought stress imposed through PEG-6000 substantially impaired seed germination and early seedling growth across maize genotypes, which is consistent with earlier reports highlighting that osmotic stress limits seed water uptake, delays metabolic activation, and reduces radicle emergence (Liu et al., 2015; Queiroz et al., 2019; Mustamu et al., 2023). The ~28%–30% reduction in germination observed under PEG stress corroborates findings in wheat and rice, where water-deficit conditions similarly delayed germination and hampered seedling vigor (Zhang et al., 2020; Li et al., 2025). Seed priming with GA_3_ partially alleviated the inhibitory effects of drought in a dose-dependent manner. GA_3_ at 100 ppm significantly enhanced germination percentage and rate, shortened mean germination time, and improved seedling length compared to drought stress alone. This recovery can be attributed to GA_3_’s well-established role in stimulating hydrolytic enzyme activities, such as α-amylase, which accelerates starch mobilization and energy supply during germination (Manavalagan et al., 2023; Rida et al., 2021). However, the slight decline at 200 ppm suggests that supra-optimal exogenous GA_3_ may disrupt endogenous hormonal homeostasis or trigger feedback inhibition, a phenomenon also reported in crops like maize, barley, and rice (Chen et al., 2022; Guan et al., 2019; Zhang et al., 2020; Li et al., 2025; Wang T et al., 2025). In contrast, melatonin consistently outperformed GA_3_ across most measured traits. At 200 µM, melatonin treatments restored germination percentages, water uptake, and seedling biomass close to non-stressed controls, underscoring its superior protective role. This can be explained by melatonin’s dual function as a direct ROS scavenger and as a regulator of antioxidant enzyme systems (Singh et al., 2023b). Improved water uptake observed in melatonin-treated seeds indicates melatonin-mediated regulation of aquaporin activity and osmotic adjustment, which is increasingly being recognized as a key mechanism in seed drought tolerance (Qiao et al., 2020; Arnao and Hernández-Ruiz, 2021; Jahan et al., 2023; Tiwari et al., 2020; Fu et al., 2024). Enhanced seedling length and biomass under melatonin priming further align with recent findings in maize, soybean, and wheat, where melatonin priming ameliorated drought effects by maintaining hormone balance, improving mitochondrial function, and stimulating radical detoxification pathways (Ahmad et al., 2021; Li et al., 2021; Muhammad et al., 2023; Zhang et al., 2025).

Genotypic differences observed in the present study highlight genetic variation in drought resilience. DHM 117 and CLO 2450 were more resilient, showing higher recovery in germination and biomass than SML 1. These findings mirror previous reports where hybrid maize genotypes exhibited differential responses to drought priming, primarily due to differences in intrinsic antioxidant capacities, hormonal regulation, and osmotic adjustment strategies (Zhang et al., 2025). The findings of this experiment establish melatonin as a more potent priming agent than GA_3_ for mitigating drought-induced germination and seedling inhibition in maize (Singh et al., 2023b). While GA_3_ enhanced energy metabolism and germination kinetics at optimal concentrations, melatonin provided broader protection by integrating antioxidant defense, osmotic regulation, and improved water uptake (Li et al., 2019; Shokat et al., 2023; Jurado-Mañogil et al., 2024; Franco-Navarro et al., 2025; Ismail et al., 2025).

### 4.2 Differential effects of standardized gibberellic acid and melatonin seed priming on biochemical responses of maize genotypes under drought stress

In the present study, the exposure of germinating maize seeds to 10% PEG-6000 resulted in sharp reductions in α-amylase activity, soluble sugars, proline, phenolics, and proteins, along with significant increases in lipid peroxidation and electrolyte leakage. These findings corroborate previous reports that drought-induced osmotic stress suppresses hydrolytic enzyme activity, restricts seed metabolism, and accelerates oxidative injury (Ahmad et al., 2021; Zhang et al., 2025). Seed priming with GA_3_ proved highly effective in ameliorating stress-induced declines in α-amylase activity and total soluble sugars, often exceeding control levels in genotypes SKM 1 and SML 2. These results align with earlier findings that GA_3_ promotes seed reserve mobilization by enhancing amylolytic enzyme expression and activity, thereby supporting germination under water-deficit conditions (Anjum et al., 2017; Kawaguchi et al., 2023; Mickky and Aldesuquy, 2017; Haghpanah et al., 2024). In our study, GA_3_-primed seeds maintained higher carbohydrate availability at both 7- and 14-day intervals, confirming its role in stimulating starch hydrolysis and ensuring sustained energy delivery to actively growing embryonic tissues. Such enhancement of enzymatic activity is consistent with GA_3_-mediated upregulation of carbohydrate metabolism and signaling pathways that accelerate radicle protrusion and seedling elongation under adverse environments. Melatonin priming promoted significant increases in phenolic content and maintained higher proline and moderate soluble sugar levels, particularly at later stages of germination. These traits highlight melatonin’s role in ROS detoxification and redox homeostasis, consistent with recent evidence that melatonin enhances endogenous antioxidant metabolism, reduces membrane lipid peroxidation, and boosts osmoprotectant accumulation under abiotic stresses (Arnao and Hernández-Ruiz, 2019, 2021; Gu et al., 2022). Melatonin-treated seeds exhibited lower TBARS levels and reduced electrolyte leakage, especially in stress-sensitive genotypes such as SML 163 and SML 165, confirming its superior membrane-stabilizing effect. This antioxidative protection likely arises from melatonin’s ability to directly scavenge ROS and/or stimulate antioxidant enzyme activity, as reported in maize and other cereals under PEG and drought stress (Kleszczyński et al., 2016; Jahan et al., 2019; Yu et al., 2022; Monteiro et al., 2024).

Proline accumulation is a key adaptive mechanism under osmotic stress, functioning as a compatible solute, hydroxyl radical scavenger, and stabilizer of proteins and membranes (Kaur and Asthir 2015; Siddique et al., 2018). Interestingly, drought stress significantly reduced proline levels in this study, contradicting the typical osmotic adjustment response. The decline in proline content under severe PEG-induced drought stress may be due to the suppression of proline formation within plant tissues. Under intense water deficit, extreme cellular dehydration disrupts normal metabolic activities, leading to reduced functioning of key enzymes involved in proline synthesis, particularly Δ^1^-pyrroline-5-carboxylate synthetase (P5CS) (Sharma et al., 2011). Severe stress also restricts carbon and nitrogen metabolism, thereby limiting the supply of glutamate, which is the main precursor for proline production. Moreover, PEG-induced stress can increase oxidative damage and impair the functioning of mitochondria and chloroplasts, causing a shift in cellular priorities from osmolyte accumulation to basic survival and repair processes. In some cases, enhanced breakdown of proline through proline dehydrogenase (ProDH) may further lower proline levels. Therefore, the observed reduction in proline content indicates a breakdown of normal metabolic regulation rather than an effective osmotic adjustment response under severe drought conditions (Bhaskara et al., 2015). GA_3_ priming strongly enhanced proline accumulation in stress conditions, exceeding control levels in SKM 1 and SML 2, suggesting GA_3_’s potential in stimulating proline biosynthesis via hormonal crosstalk and redox-mediated pathways. Melatonin also improved proline content, although moderately, consistent with its role in stabilizing osmotic homeostasis gradually rather than stimulating rapid accumulation (Li et al., 2021). A notable observation was the enhancement of soluble protein content in GA_3_-primed seeds, reaching peaks >56 mg g^−1^ FW in SKM 1, LM 13, and SML 2. This indicates that GA_3_ stimulates stress-induced *de novo* protein synthesis, possibly associated with late embryogenesis abundant (LEA) proteins, antioxidant enzymes, and enzymes of carbohydrate metabolism (Magwanga et al., 2018). Similar observations were reported by Jaybhaye et al. (2024) in soybean and Huang et al. (2025) in rice, linking GA-dependent signaling to proteomic reprogramming under drought. However, melatonin induced moderate but sustained increases in protein content, pointing to its role in protecting existing proteins from oxidative degradation, consistent with its function in protein stabilization and post-translational modification under oxidative conditions (Ghatak et al., 2017; Arnao and Hernández-Ruiz, 2021).

Stress-induced elevations of TBARS in drought-treated seeds reflected enhanced lipid peroxidation, a classical marker of oxidative stress in germinating seeds (Mittler, 2017). Interestingly, GA_3_ priming further elevated TBARS levels, suggesting that the GA-induced intensification of metabolic activity also accentuated ROS accumulation, possibly due to increased mitochondrial respiration and accelerated metabolism (Guo et al., 2017; Al-Harthi et al., 2021; Kaur et al., 2023). Melatonin, in contrast, limited TBARS accumulation, especially in SKM 1 and SML 176, thereby effectively reducing peroxidative damage to membranes. Moreover, electrolyte leakage data strongly supported melatonin’s protective role, as it consistently displayed lower leakage (mean 79.47%) compared to GA_3_-primed seeds (82.17%), highlighting its superior efficiency in maintaining cell membrane integrity under stress. These findings agree with recent evidence reporting melatonin as a key regulator of stress-induced oxidative membrane injury in maize and rice (Huang et al., 2019; Yan et al., 2021). Marked genotypic variation was observed in response to priming treatments, with SKM 1, SML 2, and LM 13 exhibiting high resilience across multiple biochemical parameters, while SML 163 and SML 165 appeared more stress-sensitive. Such differential responses emphasize the inherent genetic variability in stress perception, signaling, and metabolic reprogramming among maize genotypes (Malenica et al., 2021; Yang et al., 2023).

## 5 Conclusions

The present study elucidates the differential yet complementary roles of GA_3_ and melatonin in mitigating drought stress during maize germination, offering valuable insights into agronomic interventions in water-scarce regions. Our findings reveal that GA_3_ priming predominantly augments hydrolytic metabolism, enhances reserve mobilization, and promotes the accumulation of proline and proteins, thereby facilitating early seedling vigor under stress conditions. In contrast, melatonin supports osmotic adjustment and maintains membrane stability, underscoring its role in long-term stress resilience. However, further investigation on the effects of gibberellic acid and melatonin treatment on maize growth and yield under drought stress conditions will help develop a more comprehensive approach to improve maize tolerance to drought stress.

## Data Availability

The original contributions presented in the study are included in the article/Supplementary Material. Further inquiries can be directed to the corresponding authors.
